# Mobile technology intervention to improve care coordination between HIV and substance use treatment providers: development, training, and evaluation protocol

**DOI:** 10.1186/s13722-017-0073-1

**Published:** 2017-03-14

**Authors:** Kasey Claborn, Sara Becker, Susan Ramsey, Josiah Rich, Peter D. Friedmann

**Affiliations:** 1 Department of Medicine, Division of General Internal Medicine, Rhode Island Hospital, 111 Plain Street, Providence, RI 02903 USA; 2 The Warren Alpert Medical School, Brown University, Box G-BH, Providence, RI 02912 USA; 3 Center for Alcohol and Addiction Studies, Brown University School of Public Health, 121 South Main Street, Box G-121-5, Providence, RI 02912 USA; 4 Miriam Hospital, Providence, RI 02906 USA; 5 Office of Research, Department of Medicine, University of Massachusetts – Baystate and Baystate Health, Springfield, MA USA

**Keywords:** HIV, Addiction, Communication, Mobile technology, Treatment providers

## Abstract

**Background:**

People living with HIV (PLWH) with a substance use disorder (SUD) tend to receive inadequate medical care in part because of a siloed healthcare system in which HIV and substance use services are delivered separately. Ideal treatment requires an interdisciplinary, team-based coordinated care approach, but many structural and systemic barriers impede the integration of HIV and SUD services. The current protocol describes the development and preliminary evaluation of a care coordination intervention (CCI), consisting of a tablet-based mobile platform for HIV and SUD treatment providers, an interagency communication protocol, and a training protocol. We hypothesize that HIV and SUD treatment providers will find the CCI to be acceptable, and that after receipt of the CCI, providers will: exhibit higher retention in dual care among patients, report increased frequency and quality of communication, and report increased rates of relational coordination.

**Methods/design:**

A three phase approach is used to refine and evaluate the CCI. Phase 1 consists of in-depth qualitative interviews with 8 key stakeholders as well as clinical audits of participating HIV and SUD treatment agencies. Phase 2 contains functionality testing of the mobile platform with frontline HIV and SUD treatment providers, followed by refinement of the CCI. Phase 3 consists of a pre-, post-test trial with 30 SUD and 30 HIV treatment providers. Data will be collected at the provider, organization, and patient levels. Providers will complete assessments at baseline, immediately post-training, and at 1-, 3-, and 6-months post-training. Organizational data will be collected at baseline, 1-, 3-, and 6-months post training, while patient data will be collected at baseline and 6-months post training.

**Discussion:**

This study will develop and evaluate a CCI consisting of a tablet-based mobile platform for treatment providers, an interagency communication protocol, and a training protocol as a means of improving the integration of care for PLWH who have a SUD. Results have the potential to advance the field by bridging gaps in a fragmented healthcare system, and improving treatment efficiency, work flow, and communication among interdisciplinary providers from different treatment settings.

Trial Registration: NCT02906215

## Background

Alcohol and illicit drug use remains a substantial problem among people living with HIV (PLWH), contributing to suboptimal treatment outcomes and increased transmission of HIV. Over 81% of PLWH report use of an illicit drug, and nearly one in four meet diagnostic criteria for a substance use disorder (SUD) [1]. Substance use is associated with poorer outcomes across the HIV care continuum including delayed diagnosis and linkage to HIV care, decreased adherence to antiretroviral medications, reduced retention in care, increased sexual risk behavior leading to inferior clinical outcomes, and increased HIV transmission and drug resistance [2]. HIV-infected people who use drugs are more likely to have medical (e.g., hepatitis C, tuberculosis) and psychiatric (e.g., depression, anxiety) comorbidity, neurocognitive impairment [3], and increased risk for drug overdose [4], resulting in increased utilization of services, morbidity, and mortality [5–7]. Further, this population tends to cycle in and out of treatment, resulting in suboptimal health outcomes [8, 9]. PLWH who have a SUD tend to receive inadequate medical care [10] which is in part a byproduct of traditional HIV care and substance use services being delivered separately [11].

The Institute of Medicine has recommended integrated care between substance use disorder (SUD) treatment and primary care since 2006 [12, 13]; however, there remains a dearth of evidence in regards to the optimal method of service integration. Both HIV-infection and SUDs are chronic and treatable diseases requiring continued care [13]. With higher rates of SUDs among HIV-infected populations [2], early identification of SUDs and referral to treatment is vital to effective clinical management and prevention of HIV transmission. An interdisciplinary, comprehensive, team-based coordinated care approach has been identified as the ideal treatment for PLWH who have a SUD [13]. Benefits of service integration have been noted at the patient-, provider-, and societal-levels, including decreased healthcare costs and improved treatment outcomes [14–16]. Ideal treatment includes an interdisciplinary team-based approach which can improve tracking and monitoring of patients, and coordination of comprehensive treatment plans, which may prevent treatment drop-out or expedite patient re-engagement in care [16].

Many structural and systemic barriers impede the integration of treatment for HIV and SUDs. Start-up costs and limited building space are frequent deterrents of such integration. Consequently, patients with comorbid diagnoses tend to be referred to multiple providers at off-site clinics and are often lost in the referral process. Further, effective integration and quality treatment require additional staff and provider training in both HIV disease and substance use treatment [17]. Montague et al. [18] identified several gaps in care among 119 HIV treatment providers and 159 SUD treatment providers. Results demonstrated that providers need cross-training to increase knowledge of risks associated with these comorbid conditions and require training in effective assessment and identification for both diseases. Additional training needs included education about available referral sources for dual treatment and the need to define the role of SUD treatment providers in supporting HIV care [18]. Co-management of HIV and SUDs hinges on effective communication between a team of interdisciplinary providers. Current infrastructure frequently limits timely information transfer, resulting in deficits in communication. Further, cultural and language differences within the independent organizational structures and provider-related stigma are important barriers to integrating HIV and SUD treatment [19]. There exists an urgent need for the healthcare infrastructure to adequately address the needs of dually diagnosed patients in a cost-effective and easily implemented manner. Information technology solutions designed for providers may address many of these barriers to dual care.

Rudin and Bates [20] identified four areas within a care coordination framework that would likely benefit from information technology solutions: (1) the ability to identify a patient’s care team both within and across settings and disciplines (including primary care providers, specialists, social worker, case manager, substance use counselor), (2) the ability to collaborate with the care team in a quick and efficient manner, (3) the ability to collaborate through the sharing and formulation of care plans, and (4) the ability to monitor and track task responsibilities [20]. However, electronic health record (EHR) systems still lag in several areas including: information exchange across settings; development, storing, and sharing of care plans; tracking of referrals; and improved team-based communication [21].

A preliminary study conducted by the investigative team highlighted HIV and addiction treatment provider perspectives in regards to care coordination barriers and strategies to improve coordination [22]. In-depth qualitative interviews revealed concerns that EHRs, as they are currently designed, may result in poorer comprehensive care for PLWH who have a SUD as a result of stigma related to the documentation of both HIV disease and substance use. Providers expressed a desire for development of an integrated, secure technology platform specifically designed for HIV and addiction providers as a solution to improved communication and care coordination across disciplines. Recommended features included an instant messaging system, identification of the patient’s care team and contact information, real-time notifications, HIV and SUD training resources, and community resources. Providers conveyed interest in a range of potential delivery modalities including a web-based platform and mobile platforms accessed through tablet or mobile phone devices. When asked explicitly what platform they would prefer, a majority of providers preferred a tablet- or phone-based mobile platform, due to the perceived benefits of increased mobility provided by these devices. Additional perceived benefits included the capability to interact with patients with the technology interface on a tablet device during appointments and potential for features to allow real-time notifications and easy access to data (e.g., have alarms for notification if a high-risk patient missed appointments or is admitted into the emergency department). Some providers noted, however, that they would most value a mobile platform if it could either be co-located (i.e., stored on the same workstation) or fully integrated (i.e., embed within) the EHR in order to minimize duplication of data entry, highlighting the need for a tool that can be flexibly delivered to meet provider preferences.

Mobile health technology offers a unique potential platform for rapid communication among providers and tracking of patient service utilization and treatment approaches. Additionally, it has the potential to improve the team-based approach consistent with the collaborative care model, while promoting more effective care in hard-to-retain populations that place a significant burden on the healthcare system, such as PLWH who have a SUD. Currently, there are over 200 HIV-related mobile health applications marketed for PLWH on either android or apple platforms. A 2013 review of mobile applications found that only 55 apps promoted HIV prevention and care services, and the majority of these apps focused on providing disease-specific educational information only [23]. No mobile applications were identified for *treatment providers*. Mobile applications are unique among devices and tools used in clinical practice in that there is currently no mechanism for regulating them or ensuring they are efficacious or beneficial. A theory-driven and empirically tested technology platform for providers that over the long-term be flexibly delivered as either a stand-alone product or integrated with the EHR has the potential to improve coordination and treatment management of dually diagnosed patients.

**Fig. 1:**
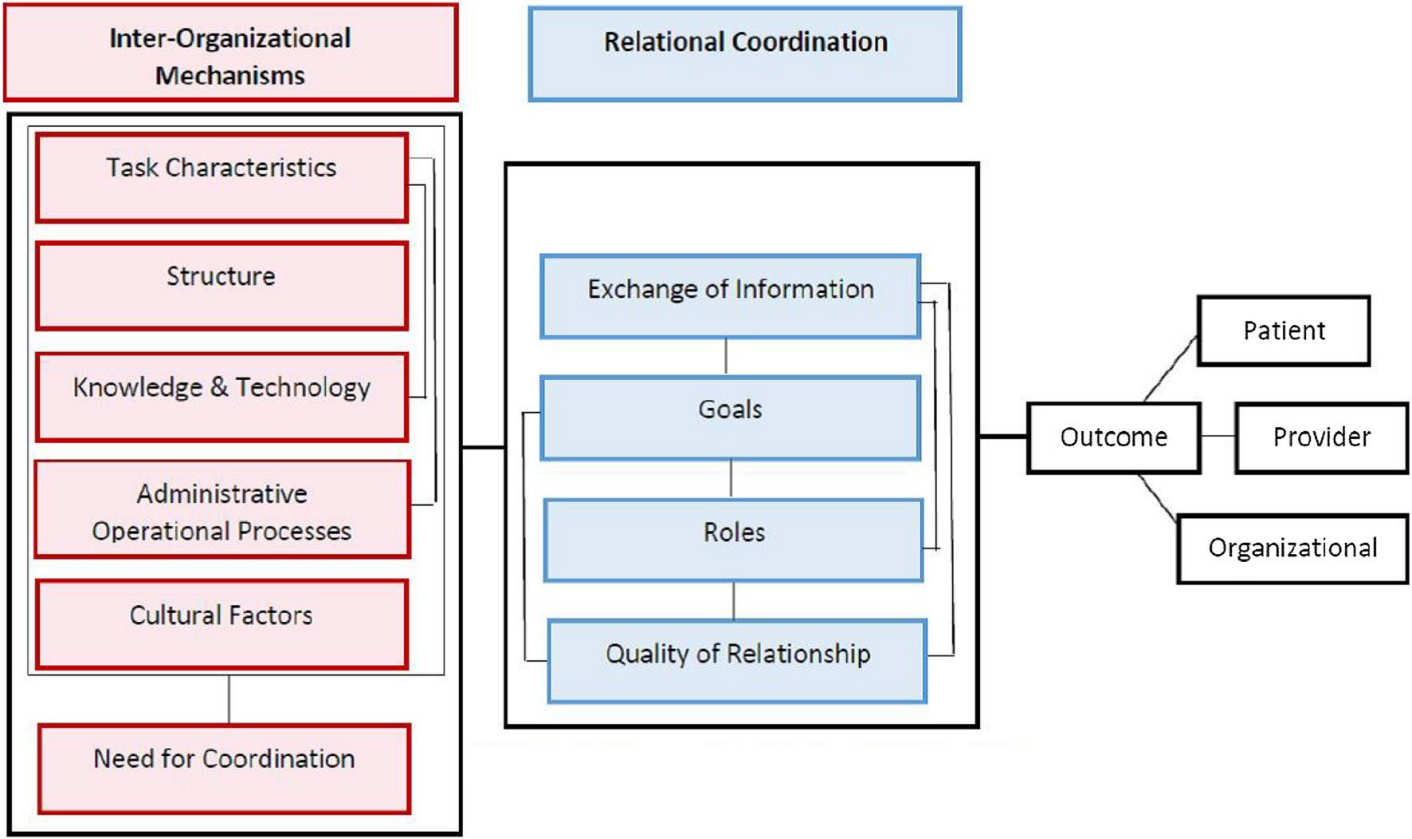
Care coordination framework adapted from Van Houdt et al. [25]

For the purposes of this study, care coordination is defined as “the deliberate organization of patient care activities between two or more treatment settings [HIV clinic, SUD clinic] involved in a patient’s care to facilitate the appropriate delivery of health care services; organizing care involves the marshalling of personnel and other resources needed to carry out all required patient care activities and is often managed by the exchange of information among participants responsible for different aspects of care” (p. 2) [24]. Van Houdt et al. [24, 25] developed a theoretical framework of care coordination which encompasses organizational-, provider-, and patient-level characteristics (see Fig. 1). This framework will guide the development of a technology-assisted care coordination intervention (CCI) for HIV and substance use treatment providers. Consistent with the Care Coordination framework, the CCI seeks to improve relational coordination components through modification of the inter-organizational mechanisms of structure, knowledge and information technology, administrative operational processes, and cultural factors. A wealth of literature has demonstrated that didactic training-alone is insufficient to produce optimal change [26, 27]. Thus, we will use an evidence-based comprehensive training approach to promote adoption of the CCI that addresses both organizational and individual provider level factors [28–30].

### Present study

This study uses mixed methods to optimize care coordination of patients diagnosed with HIV and substance use issues using an organizational-level intervention that combines an evidence-based training approach with use of mobile technology. The proposed project consists of three phases. In Phase 1, we will use qualitative data to guide the development of the CCI and training approach. Phase 2 will consist of a series of functionality tests of the tablet-based mobile application prototype using fake patient data with HIV and SUD treatment providers, and a review of the intervention with a stakeholder panel of clinic leadership. Data regarding feasibility, acceptability, and barriers that would limit effectiveness of the intervention will guide modifications to finalize the software and the training protocol. In Phase 3, we will conduct a pre-, post-test trial of the CCI to examine its effects on the amount and quality of interagency communication, perception of interagency professional relationships, and retention of patients in dual care. Preliminary data regarding intervention costs and sustainability potential will be collected. Table 1 depicts the timing of key protocol elements for the Phase 3 pilot trial including provider enrollment, provider training, and assessments.

**Table 1:** Timing of provider enrollment, training, and assessment activities

Timepoint	Enrollment	Training	Post-didactic training			
	-t1	0	Post training	+1 month	+3 months	+6 months
*Provider enrollment*						
Informed consent/assent	X					
*Training*						
Didactic sessions #1–3		X				
Interagency communication manual		X				
Internal change champions			X	X	X	X
External leadership coaching			X	X	X	X
*Assessments*						
Provider-level outcomes	X		X	X	X	X
Organizational-level outcomes	X			X	X	X
Patient-level outcomes	X					X

The primary goal of this protocol is to develop and test a CCI among HIV and SUD treatment providers. We hypothesize that the combination of training and mobile health tools specifically created for HIV and SUD care coordination will (a) increase patient retention in dual care, (b) increase the amount of communication between HIV and addiction treatment providers, and (c) improve relational coordination between HIV and SUD treatment providers.

## Methods

### Participants and setting

Two organizations in the Northeast region of the United States, an academic affiliated HIV clinic and a non-profit SUD treatment facility, will serve as the recruitment and implementation sites for the CCI. The HIV clinic is funded by the Ryan White program and provides comprehensive care for over 1600 HIV-infected patients. The clinic also has a pre-exposure prophylaxis (PrEP) program which offers a single, daily pill to patients at higher risk for HIV exposure to prevent the person from being infected with HIV. The collaborating SUD treatment program is a non-profit organization that provides substance use treatment, recovery, and prevention services to about 950 new patients annually across four clinics. These services include general outpatient (drug free) or intensive outpatient counseling for alcohol and drug-related problems.

Key stakeholders (n = 8 in Phase 1: stakeholder interviews) and frontline HIV and SUD treatment providers (n = 8 in Phase 2: functionality testing; and n = 60 in Phase 3: Pilot Trial) will be recruited from the HIV and SUD clinics. Upper management in each organization will inform providers that participation in the study is available and they may volunteer to participate; providers will be explicitly informed that participation is voluntary and will not affect their employment. Once a potentially eligible provider has been identified, the RA will contact the provider, introduce the study, and assess eligibility. For inclusion, key stakeholders must: (a) be at least 18 years of age, (b) have a major administrative or leadership role (i.e., President/CEO, Clinic Director, Executive Leadership) in an HIV or SUD treatment center, (c) have been employed in their current leadership position for at least 6 months, and (d) able and willing to provide informed consent. Treatment providers must: (a) be at least 18 years of age, (b) provide either HIV care or SUD treatment [e.g., physicians, residents, psychologists, mid-level providers], (c) have been employed at a participating recruitment site for at least 6 months, (d) have no plans for a leave of absence over the next 2 years and (e) willing and able to provide informed consent. We will recruit an even number of stakeholders and providers from HIV and SUD treatment centers. We recognize that staff turnover may occur throughout the study and especially during Phase 3 (6-month intervention phase). Providers who leave employment during the trial period will be asked to return the tablet device to the study and will be removed from participating in the CCI for the remainder of the study. Participants in Phase 1 (stakeholder interviews) will receive $75, and participants in Phase 2 (functionality testing) will be paid $50. In Phase 3 (pilot trial), participants will be paid $50 for completion of the baseline interview and $40, $45, and $50, respectively, for completion of the 1-, 3- and 6-month follow-ups. Participants completing exit interviews will receive an additional $50.

### Procedures

#### Phase 1: qualitative methods to inform intervention development

Individual interviews with 8 key stakeholders and clinic audits will be used to inform development of the CCI. All qualitative interviews will follow a semi-structured agenda covering the following topics: (a) interagency processes that facilitate or impede interagency collaboration; (b) organizational resources available to support interagency collaboration; (c) professional knowledge domains and boundaries (i.e., substance use providers knowledge of HIV care and vice versa); (d) expectations in regards to authority to coordinate treatment; (e) issues of patient confidentiality in regards to coordination of HIV and SUD care. Interviews will last approximately 60 min, and participants will receive $75. We will regularly assess data saturation on key topics and may conduct a higher number of interviews if more information is required. In addition, the CCI will be informed by 29 individual interviews with HIV and SUD treatment providers that were conducted as part of another NIDA funded study (K23DA039037). The goal of this phase is to gather organizational-level data to evaluate potential barriers and facilitators to collaboration within each clinic setting.

##### Audit of referral and communication process

To augment the stakeholder interviews, audits within each clinic will be conducted to assess existing referral and communication procedures. Data to be extracted include number of relevant referrals by provider type, number of patients followed post-referral, and language used in referrals and consult letters. These data will inform the interagency communication protocol and language for the CCI.

##### Coding and analysis

Qualitative data will be analyzed using a thematic framework method [31]. This method will allow the investigative team to develop themes from both the research questions and the narratives of the research participants [32]. A preliminary coding structure will be derived deductively from the interview script, with specific subtype coding applied inductively as themes and repetitions emerge from the data. Data analysis will be iterative using standard analysis techniques, including open coding, axial coding, marginal remarks, and memo-writing [33]. Preliminary research questions include: (1) what are the gaps in existing referral and communication processes; (2) how do providers exchange patient information; (3) how do providers perceive the need for care coordination; and (4) what is the level of cross-training and available resources.

#### Phase 2: intervention and manual development & refinement

Based on data triangulated from Phase 1 and evaluation of the literature, we will develop the CCI prototype and refine the training and intervention protocols. The development of the CCI will involve a series of meetings among the investigators and the software development team to review the core elements and features of the mobile application. The goal of these meetings will be to match the intervention content and structure to the needs and preferences identified through formative research.

##### Intervention

The CCI will consist of a mobile application developed for treatment providers, an interagency communication protocol, and a training protocol. The mobile application will be developed on a secure portable platform that providers will access through study-provided tablet devices. The CCI dashboard will be user friendly and easily navigable. The application will feature a secure messaging center for providers to directly communicate. Other application features will include: (a) resources for methods of identifying patients in need of dual care, (b) a system to refer patients to treatment, (c) ability to track patients’ access and utilization of services, (d) real-time patient care notifications, (e) identification of a patient’s care team, and (f) up-to-date lists of resources and evidence-based recommendations for treatment options. The CCI will provide a secure platform that will enable the transfer of patient health information between providers, yet will not be linked in the same electronic health record system, adding an additional level of privacy for patients who do not wish for non-HIV/SUD providers to have access to this sensitive data. These features of the CCI prototype are based on preliminary work [22]; however, the final app will reflect data gathered from the Phases 1 and 2 of the study.

##### Refinement of the mobile application and training materials

Once the prototype CCI and training materials have been developed, these materials will be refined through an iterative process whereby we will test the functionality of the CCI with 6–8 providers. During these individual interviews, we will demonstrate the CCI features and solicit feedback on the training procedures and the interagency communication protocol. These data will inform modifications to the CCI. Once modifications have been completed, the investigative team will have a 2-h (in-person/teleconference/Skype) meeting with the 8 stakeholders who participated in Phase 1 to review modifications and finalize the CCI.

#### Phase 3: provider training and intervention testing

Phase 3 will test the organizational intervention. We recognize use of the mobile application alone is likely to yield minimal improvements in care coordination. Consequently, the proposed intervention augments the mobile application with training of providers and on-going support for training needs.

##### Training procedures

Although the primary aim of the proposed study is intervention development, we are mindful of the need to have an evidence-based replicable training protocol to facilitate widespread implementation. We use the Service to Science Laboratory (SSL) staff training model, which was specifically developed by the SAMHSA-funded Addiction Technology Transfer Centers (ATTCs) to increase support for intervention adoption at both the individual-level and organizational-level. The SSL model contains three key elements: upfront didactic training, ongoing performance feedback, and ongoing coaching from an external leadership coach who works with Internal Change Champions within each organization [34].

The upfront didactic training will be led by a member of the New England ATTC staff and will consist of three training sessions (see Table 2 for an overview of topics). Participants will complete a 20-item baseline knowledge assessment prior to Training Session #1. All training sessions will be audio-recorded and participants who are unable to attend will be required to listen to the audio recording and attend a brief make-up training session. Upon completion of Training Session #3, each treatment provider will be required to complete a 20-item post-training knowledge assessment and to demonstrate adequate knowledge, defined as accurate answers to 16 (80%) of the items. Each provider will also complete a role play with the ATTC trainer using a standardized patient case. During the role play, the provider will be required to demonstrate competence in the following six areas: (a) identifying resources to detect patients in need of dual care, (b) using the referral system (including ability to both make a referral and accept a referral request), (c) tracking of patients’ access and utilization of services, (d) reading real-time patient care notifications, (e) identifying a patient’s care team, and (f) reviewing up-to-date lists of resources and evidence-based recommendations for treatment options. Providers who do not demonstrate competence will receive immediate corrective feedback and up to two additional role plays will be conducted until competence is shown. Only those providers who exhibit competence in all six areas will proceed to the next phase of training.

**Table 2:** Expected features and benefits of the care coordination intervention (CCI) based on preliminary qualitative work

Training procedures	
Interagency communication protocol	Include a manual for the mobile app and outline communication procedures and language
Didactic Session 1 (conduct separately at each clinic)	Provide study details, solicit buy-in and commitment to change, cross-training in HIV/SU issues
Didactic Session 2 (conduct concurrently at neutral location)	Train in CCI and study procedures, introduce participants from each clinic
Didactic Session 3 (conduct separately at each clinic)	Hands-on practice with the mobile application using fake patient data, pass a CCI competency test
Science to Service Laboratory (ATTC)	Appoint Internal Implementation Champions within each clinic; external leadership coach

Mobile application features				
Enable rapid communication	Patient linkage to dual care	Care coordination	Work flow	Dual care resources
Portable platform	Identification of patients in need	Identification of care team	Direct referrals from providers	Patient educational resources
Secure messaging center	Easy referral system	Easily develop dual care plans	Manage transitions to dual care	Provider educational resources
Notification of message receipt	Track patient’s service usage/appointment attendance	Communicate with care team via SMS procedures	Decrease time spent on coordinating care	Available community resources and admission criteria
Remote access to system	Link to HIV/PrEP/SU services	Track and manage dual care	Decision trees for referrals	Identify provider availability

After completion of the 3-session didactic training series, we will enable the mobile application, assign each provider a tablet device, and initiate the 6-month open trial. At this point, the SSL training model will shift from a focus on didactics to ongoing interpersonal support. The SSL relies heavily on supportive interpersonal personal strategies through the use of an external leadership coach and Internal Change Champions, who continually encourage collaborative problem solving throughout the organization. The eight key stakeholders will be responsible for either serving as Internal Change Champions or selecting alternates. The external leadership coach will have monthly coaching calls with the Internal Change Champions focused on tracking implementation progress and engaging in problem solving to address barriers to sustained use of the mobile application. In turn, the Internal Change Champions will be responsible for providing ongoing supervision and support to frontline treatment providers. Consistent with the recommendations of Montague et al. [18], who noted the need for cross-training among HIV and substance use providers, the SSL will include cross-training via explicit didactic instruction and ongoing coaching in an effort to increase understanding of each provider’s role and disease specific processes.

##### Measures

The framework identified in Fig. 1 has informed the battery of measures. Assessments will focus on organizational-, provider-, and patient-level domains (see Table 3). Primary outcomes will be feasibility and acceptability of the CCI and assessment procedures to be used in a larger trial [35]. Organizational outcomes will include pre- and post-intervention assessments of each clinic to gather data on organizational structure and procedures for care coordination. Provider outcomes will include feedback on the intervention component in Phase 1 and 2 of the trial; satisfaction of care coordination, frequency and quality of interagency communications, and interagency professional relationships; and potential moderators (e.g., stigma, perceived coercion). Patient outcomes will include demographics, viral load, and retention in dual care. Quantitative measures will be collected via web-based surveys from clinic leadership (n = 8) and providers (n = 60) at baseline and 1-, 3-, and 6-months post implementation of the CCI (Phase 3).

**Table 3:** Outcome variables and assessment points

Quantitative measures	BL	Post-T	1-, 3- mos	6 mos	Analysis
Provider-level outcomes					
Provider descriptive information					
Provider demographics	X				Descriptive
Training in HIV and SU issues	X		X	X	Descriptive
HIV/SU treatment experience	X		X	X	Descriptive
Knowledge related to SU/HIV	X	X		X	Descriptive
Coercion assessment scale	X		X	X	Exploratory: Moderator
Provider perception inventory	X		X	X	Exploratory: Moderator
Provider acceptability and usability of the CCI and training protocol					
Satisfaction with training		X			Acceptability
CCI program satisfaction				X	Acceptability
Perceived attributes scale	X		X	X	Exploratory: Mediator
Interagency collaboration and communication					
Levels of collaboration scale*	X		X	X	Quant: Hyp 2
Frequency/quantity of communication*	MDE		MDE	MDE	Quant: Hyp 2
Wilder collaboration factors inventory	X			X	Exploratory: Moderator
Relational coordination					
Relational coordination scale*	X		X	X	Quant: Hyp 3
Organizational-level outcomes					
Organizational descriptives	X			X	Descriptive
Organization readiness	X		X	X	Exploratory: Mediator
Implementation climate scale	X		X	X	Exploratory: Mediator
Patient-level outcomes					
Descriptive information					
Demographics	MDE				Descriptive
Viral load	MDE			MDE	Descriptive
Dual care treatment retention	C/MDE			C/MDE	Quant: Hyp1

*BL* baseline, *I* implementation, *T* training, *MDE* Data extracted through the mobile app dashboard, *CDE* data extracted through clinic medical records, *EXPL* exploratory, *QUANT* quantitative, *HYP* hypothesis

* Primary Outcome Variable

##### Provider-level descriptives

The following basic demographic and clinical experience data will be collected from providers enrolled in the study at baseline: age, gender, race, ethnicity, educational attainment, employment status, position title, number of years in practice, level of experience treating patients with HIV and substance use, knowledge of HIV and substance use treatment, and prior training experience. Providers will also complete measures indicating their willingness to participate in the study and their perceptions of stigma related to dual care. The *Coercion Assessment Scale* (CAS) [36, 37] is an 8-item measure of voluntariness to participate in research. The *Provider Perception Inventory* [38] is a 39-item scale that measures health service providers’ stigma in regards to HIV, substance use, and MSM behavior, which will be used to measure provider-related stigma.

##### Provider acceptability and usability of the CCI

We will measure providers’ perceived acceptability of and satisfaction with the CCI, as well as their perceptions of the intervention. Provider acceptability data will be gathered primarily through qualitative exit interviews at the 6- month follow-up. Providers will also be asked to complete a program satisfaction survey which will assess level of satisfaction for each component of the CCI. Perceptions of the intervention will be measured via the Perceived Attributes Scale, a 27-item, customizable scale that measures five aspects of an intervention or innovation (i.e., Relative Advantage, Compatibility, Complexity, Trialability, Observability) that have been shown to influence adoption.

##### Interagency collaboration and communication

CCI usage will be measured via the mobile application dashboard throughout the study. The Levels of Collaboration Scale [39] will be adapted for the purposes of this study and will measure progress over the five levels of collaboration and their characteristics (networking, cooperation, coordination, coalition, collaboration). The Wilder Collaboration Factors Inventory [40] is a 40-item measure that assesses strengths and weaknesses within organizations shown to be important for successful collaborations. Factors include: collaboration history, organizational climate, mutual respect, definition of roles, adaptability, and shared goal setting.

##### Relational coordination

A Relational Coordination Scale will be developed by the investigative team as there are no available measures assessing these constructs. This measure will include items assessing the following constructs: definition and awareness of provider roles in the co-management of HIV and substance use; quality of relationships between clinic providers (e.g., mutual respect, collaboration); exchange of information (e.g., transfer of information, ideas, and opinions); and setting and sharing of common goals.

##### Organizational-level

We will evaluate organizational-level descriptive characteristics as well as putative organizational mediators of CCI usage. Descriptive organizational data from participating clinics will be collected during both pre- and post-implementation site visits and include (a) number of patients at each clinic diagnosed with comorbid HIV/SUD enrolled in care; (b) number of patients receiving care at the SU clinic who are referred to testing and number tested for HIV, number of patients referred to HIV care or PrEP; (c) number of patients receiving care at the HIV clinic who are assessed for a substance use problem and number referred for substance use treatment; (d) number of patients receiving co-management care of HIV and substance use. At the 6-month post-implementation assessment, will measure also measure CCI Reach by tracking the percentage of eligible providers who participated and reasons providers chose not to participate. We will measure Organizational Readiness to Implement Change and Implementation Climate as putative mediators of CCI adoption at baseline, 1, 3, and 6 months. The Organizational Readiness for Implementing Change (ORIC) [41] questionnaire is a 12-item measure on a five-point scale, 1 (“disagree”) to 5 (“agree”). This scale will be administered to providers and key stakeholders to determine the extent to which there is a shared perception of organizational readiness for the CCI. The Implementation Climate Scale [42] will measure the degree to which there is a strategic organizational climate supportive of intervention implementation. This is a 38-item questionnaire that is scored on a five-point scale, 0 (“not at all”) to 4 (“very great extent”).

##### Preliminary assessment of implementation context

Although this study is an intervention development study, the mixed-methods design will allow us to gather preliminary data in regards to implementation context and information transfer that inform future multisite trials. We will conduct individual qualitative interviews with 4–5 key stakeholders at 6 months following implementation of the CCI. These interviews will gather data regarding perceptions of providers and leadership concerning barriers and facilitators to CCI adoption, tools needed to deliver the intervention consistently, resources needed to maintain the CCI long-term, and adaptations needed to integrate the CCI into regular practice. Qualitative data from exit interviews will be triangulated to examine themes related to barriers and facilitators of CCI adoption, stakeholder and organizational satisfaction with the CCI, and acceptability of the CCI. Additionally, we will measure the following costs associated with CCI implementation: training hours (study staff and provider time) and equipment and other resources costs.

##### Patient outcomes

Patient information will be de-identified for the study, and the research team will not have access to any private health information. Patient outcomes will be extracted through the mobile dashboard and medical chart review. No patients will be contacted for the purposes of this intervention development study; however, we recognize the importance of assessing patient outcomes in future trials. De-identified patient demographic data including age, gender, sexual orientation, race, ethnicity, educational attainment, employment status, HIV status and length of time since diagnosis, viral load, and type of substance use problem will be extracted through the dashboard on all patients entered into the system by providers. Dual care retention data will be extracted through the dashboard in regards to (a) number of appointments attended at the HIV and substance use clinics, (b) number of appointments missed, and (c) number of patients who were not retained in care.

### Data analysis plan

#### Preliminary analyses

Considering this is a pilot study, analyses will have the goal of establishing feasibility and estimation of effect sizes, with modest expectations for rejection of null hypotheses. This is especially true regarding analyses containing higher order effects and multiple predictors. Formal quantitative data analyses will be conducted only on those subjects recruited during Phase 3 of the project. Preliminary analyses will include data cleaning and examination of key demographic and baseline variable differences between the HIV providers and the substance use providers. These analyses will be exploratory in nature and used to describe the providers in each clinic. We will summarize variables at both the organizational and provider unit of analysis. Other preliminary analyses will include examinations of patterns of missing data, research dropout rates, distributional properties of dependent and other measures, and correlations among outcome measures.

#### Hypotheses testing

Given the developmental nature of this study, our primary objective is to establish feasibility and acceptability of the CCI and assessment procedures. The following deliverables will result from this study: evidence-based training model adapted to HIV and substance use providers, mobile application tool for providers targeting care coordination, interagency communication protocol, and a training strategy for the CCI. This study will yield necessary data regarding the acceptability and feasibility of the approach to be implemented in a future larger scale multisite trial [35]. We are well aware of the dangers of relying exclusively on small-scale pilot studies to gauge the promise of a novel intervention [43]. Thus, we primarily will be hoping to find a pattern of results that is supportive of the CCI rather than rigorously testing hypotheses to determine a stable effect size. Hypotheses include: (1) Compared to baseline, providers will demonstrate increased dual care treatment retention as measured by data in the EHR; (2) Compared to baseline, HIV providers will report increased frequency and quantity of communication with substance use providers, and substance use providers will report increased frequency and quantity of communication with HIV providers; and (3) Providers will report improvements in relational coordination as measured by increased scores on the Relational Coordination scale between baseline and follow-up assessment. Group means on continuous variables typically begin to stabilize around 15 participants per group. For dichotomous variables, somewhat larger sample size is needed to provide reasonably stable odds ratios for effect size estimates. Therefore, the proposed sample size of 30 participants per group, even after attrition, will allow us to evaluate the potential of the CCI intervention to improve care coordination while remaining within the budgetary parameters for a preliminary trial.

Tests of the effects of the intervention on the primary outcome variables (identified in Table 1 at BL, 1-, 3-, and 6-months) will be conducted using repeated measures ANOVAS on change scores. We will examine each organization separately and then develop a composite score to examine change across time. We will use repeated measures mixed model analysis of variance (ANOVA) to assess main effects and between-group effects on the primary and secondary dependent variables. We will test for the normality of the distributions, homogeneity of covariance matrices, and sphericity. If violations of the sphericity assumptions are found, we will use the Greenhouse Geyser Epsilon to adjust the probability of F [44]. Omnibus F testing will be followed by univariate tests to examine if primary outcome variables differ by condition (HIV vs SU provider). Analyses will be conducted using SAS Proc GLM, which allows for maximum use of observations across time even when some data are missing. To determine whether there was an effect of group, time, or a group × time interaction on the primary outcome variables specified a priori: (1) collaboration, and (2) frequency of interagency communication, and on the secondary outcome variable specified a priori: (1) interagency relationships as measured by relational coordination, we will conduct 4 repeated measures ANOVAs with 2 levels of condition (HIV provider and SU provider) and 3 levels of time (baseline, 3-mos FU, 6-mos FU). If the null hypothesis is rejected in omnibus testing, we will conduct further univariate tests of hypotheses for between-groups and main effects and calculate effect sizes for the effects observed across time (Eta squared). In addition, for each primary outcome variable at each follow up point, effect sizes and 95% confidence intervals will be computed.

#### Missing data

Although our follow-up rates in similar studies have exceeded 90%, some data will inevitably be missing. Reason for study dropout will be collected whenever possible and will be summarized. We will explore patterns of missing data to determine possible mechanisms of missingness and will apply multiple imputation techniques. We will also run a sensitivity analysis in which we will impute the outcome data for those with whom we have lost contact, using various missing data mechanisms that describe possible relationships between the study outcome and missingness. If the results of these sensitivity analyses are similar with and without the missing data, our confidence in the findings will increase.

## Discussion

Drug use remains a significant problem within the HIV pandemic. PLWH who have a SUD exhibit the highest rates of ART nonadherence among those people infected with HIV, contributing to poor treatment outcomes, increased morbidity and mortality, and HIV transmission [6, 7]. Existing treatment structures present significant challenges to adequately treating HIV disease and substance use disorders concurrently using a team-based approach among providers. This protocol seeks to improve clinical practice and treatment science by developing a combined evidence-based training and mobile application intervention for HIV and substance use disorder treatment providers. The application will be flexibly developed to enable potential future integration with the electronic health record in order to flexibly meet the needs of individual providers. This care coordination intervention will address critical inter-organizational and provider factors aimed at improving dual care and test a preliminary implementation approach.

While this study will advance our knowledge regarding approaches to care coordination among high-risk populations, a few important limitations should be acknowledged. First, this study design does not include a control condition due to the developmental nature of the protocol and to increase trial feasibility within the context of limited budgetary resources. Second, the mobile application will not be integrated with electronic health records in this initial phase, though the application will be developed to allow for integration in future versions. We chose to use a separate mobile platform that only HIV and SU providers will be able to access during this development phase for several important reasons: (a) concerns that the stigmatizing nature of HIV and SUD diagnoses might lead to inaccurate documentation in the EHR; (b) concerns about participant confidentiality since all healthcare providers have access to EHR records, and (c) concerns about the lack of EHR integration at our participating community partners, which do not currently utilize the same platform. The goal of this study phase is to develop the ideal features of the mobile application and obtain preliminary data on its acceptability, feasibility, and usability. Because EHRs and mobile technology are both rapidly evolving, we test the application as a stand-alone product in order to maximize long-term flexibility in terms of features and the potential for integration with different EHR systems. If the mobile application appears to be effective in promoting increased coordination, then the next logical question that we will evaluate in future research is whether the application is most effective when delivered as a stand-alone application, co-located with the EHR, or fully integrated into the EHR.

To our knowledge, no research to date has focused on establishing the acceptability, feasibility, and usability of mobile applications to improve care coordination with healthcare providers as the target market. Delivered over a smartphone or tablet device, such portable applications could allow for real-time patient tracking of services and on-demand access to content or services to enhance care coordination. This technology, combined with evidence-based cross-training in HIV and substance use, has the potential to overcome cracks in a fragmented healthcare system and increase clinic efficiency, work flow, and communication among interdisciplinary providers and providers of different levels. This study will produce a care coordination intervention, using combined training and mobile technology tools for providers and an organizational integration care model that can be portable to other clinics for dissemination.

## Abbreviations

CCI: care coordination intervention
TTS: technology transfer specialist
IIC: internal implementation champion
SSL: service to science laboratory
PLWHSUD: people living with HIV with substance use disorders
PrEP: pre-exposure prophylaxis
